# Supplementary material for: The Impact of Low Cardiac Output on Propofol Pharmacokinetics across Age Groups—An Investigation Using Physiologically Based Pharmacokinetic Modelling
Source: Pharmaceutics. 2022 Sep 16;14(9):1957. doi: 10.3390/pharmaceutics14091957 (PMC9502676; doi:10.3390/pharmaceutics14091957)
Supplement: Supplementary file 1 [file pharmaceutics-14-01957-s001.zip › Supplementary materials pharmaceutics PBPK CO 05082022.pdf]

SUPPLEMENTARY MATERIAL

Section 1: Methodology for optimisation and validation of the Michelet propofol model [22]

1.1 Optimization of Propofol Model

The simulations were performed using the PBPK modelling software, Simcyp® (Simcyp® Ltd, a Certara company, Version 19). An intravenous propofol model based on the published Michelet et al. model was used for simulations [22]. First, in virtual adult subjects, the clearance kinetics and volume of distribution at steady state (Vss) of the Michelet et al. propofol model were further optimised by parameter estimation of the intrinsic clearance of CYP2B6, 2C9, UGT1A9, recombinant UGT (rUGT) scalar using a Weighted Least Square (WLS) method and the Nelder-Mead minimisation approach in the parameter estimation function on Simcyp. The results of this parameter optimization are shown in Table S1.

Table S1. The Optimised Parameters Used in the Propofol Model

Parameter	Initial values*	Optimised values
**$CLint_{CYP2B6}$ (μL/min/pmol)**	21.18	39.2
**$CLint_{CYP2C9}$ (μL/min/pmol)**	0.21	0.61
**$CLint_{UGT1A9, H}$ (μL/min/pmol)**	285	360
**rUGT kidney scalar**	12.8	19.5
**Distribution model**	Rodgers-Rowland with steady-state Fick-Nernst-Planck (method 3)	Rodgers-Rowland (method 2)
**Vss (L/kg)**	12.3	16.1

**: values in the original Michelet et al. 2018 retrograde model [22]*

In a second step, the 'Paediatric' virtual population of the Simcyp simulator which contains the physiological changes in children and the ontogeny patterns of CYP2B6, 2C9, UGT1A9 , was used for all paediatric simulations. Vss was further optimised in paediatric population.

1.2 Predictive Performance and Validation

All simulations of concentration-time profiles are presented as arithmetic mean and 5-95th percentiles unless otherwise stated. Clinically observed concentration-time data used for validation were retrieving using WebPlotDigitizer v3.10 and superimposed onto simulated profiles for visual predictive checks (VPC) [23]. The observed clinical data studies used are summarised in Table S2. The VPC and 2-fold error assessment were used to determine model performance. A 2-fold prediction of observed

parameter is largely accepted though there are currently no universal consensus on the measure of predictive performance range when comparing observed data to predicted PK parameters in PBPK pharmacokinetic studies [15,24,25]. For the VPC approach, in line with the FDA Pediatric Advisory Committee, predictions are assumed to be valid if observed data points from clinical studies lie within 5th and 95th percentiles of predicted concentration–time profiles [26].

Table S2. Summary of Clinical Studies used for Validation of the Optimised Model [27-38]

Population	Clinical study	Dose	Administration	Study size	Sample matrix
**Adult**	Struys et al. [27]	2.5 mg/kg	bolus	14	plasma
	Schnider et al. [28]	2 mg/kg	bolus	8	plasma
	Levitt et al. [29]	0.1 mg/kg/min	infusion	1	plasma
	Gepts et al. [30]	9 mg/kg/hr	infusion	6	blood
	Doufas et al. [31]	200 mg	infusion	16	plasma
**Children**	Jones et al. [32]	2.5 mg/kg	bolus	12	blood
	Murat et al. [33]	4 mg/kg	bolus	12	blood
	Saint-Maurice et al. [34]	2.5 mg/kg	bolus	10	blood
**Infant**	Sepulveda et al. [35]	2.5mg/kg; 8mg/kg/hr	bolus; infusion	41	plasma
	Raoof et al. [36]	2.5 - 3.0 mg/kg *	bolus	6	blood
**Neonate**	Allegaert et al. [37]	3 mg/kg	bolus	2	plasma
	Allegaert et al. [38]	3 mg/kg	bolus	7	plasma

**: 2.75 mg/kg was simulated*

Section 2

2.1. Optimization of the Propofol Model in Adults and Children

The predicted clearance and $t_{1/2}$ of the optimised model in children and adults following the implementation of various dosing regimen of propofol as intravenous bolus or infusion were within 2-folds of the respective parameters reported in the clinical studies, except the predicted $t_{1/2}$ in Saint-Maurice et al. [34] and predicted CL in Raoof et al. [36] (Table S3). The simulated propofol concentration-time profiles recovered the respective clinically observed concentration-time data points as most of the observed data points fell within the 5th-95th percentile of the respective predicted profiles (Figure S1).

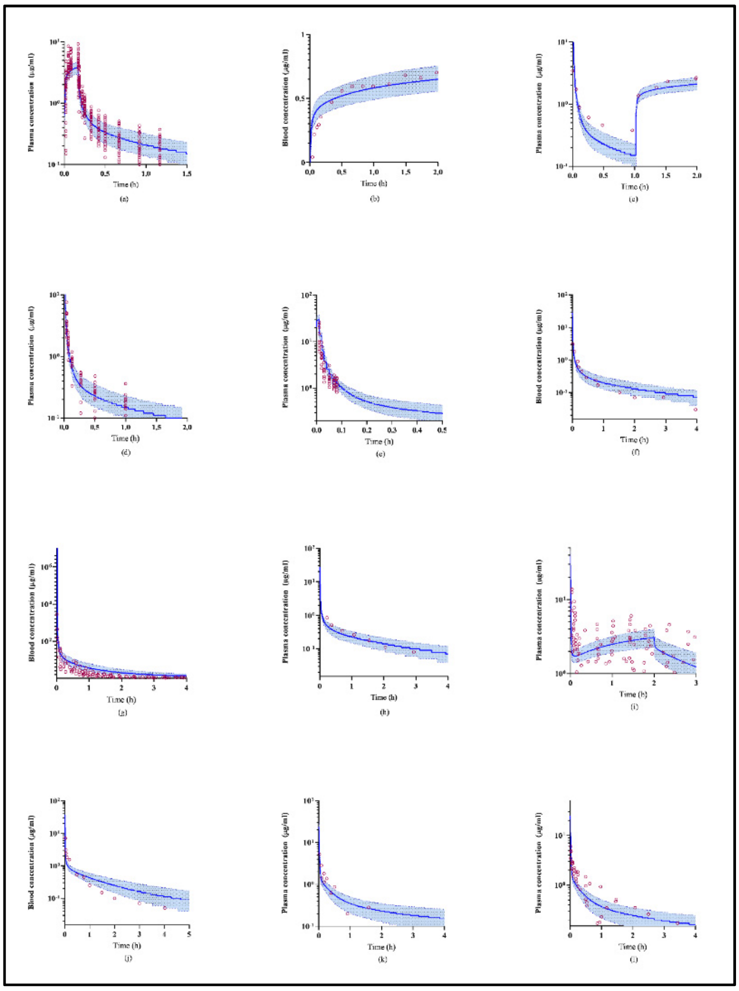

Figure S1. Simulated Plasma or Blood Concentration Time Profile of Propofol.
Dosing regimen implemented can be found in Table S2. Open red circles in (a); (b); (c); (d); and (e) represents clinically observed data points in adults retrieved from Doufas et al. [31], Gepts et al. [30], Levitt et al. [29], Schnider et al. [28] and Struys et al. [27] respectively. Open red circles in (f); (g); (h); (i); (j) and (k) represents clinically observed data points in children retrieved from Jones et al. [32], Murat et al. [33], Saint-Maurice et al. [34], Sepulveda et al. [35], Raoof et al. [36], and Allegaert et al. 2007a and 2007b [37,38]. Open red circles in (l) represent the neonatal observations. Solid lines represent population mean predictions, broken lines represent 5th and 95th percentiles of prediction and shaded green area represents predicted concentrations-time profiles within the 5th and 95th percentile.

Though there was slight underprediction of clearance of Raoof et al. [36], there was an overprediction of elimination of Levitt et al. study [29]. This discrepancy may have been due to the sample size used in Raoof (six) and Levitt (one) which may have been insufficient to capture the PK variabilities of propofol within the population [29,36]. Also, a range of administered dose (2.5 -3.0 mg/kg) was reported in Raoof et al. study while a median of that range (2.75 mg/kg) was simulated (Table S2).

Table S3. Predicted and Observed Pharmacokinetic Data of Propofol Using the Optimised Model [27-38]

Population	Clinical study	Mean ± SD Predicted		Observed	
		CL (SD) L/min	$t_{1/2}$ (SD) min	CL (SD) L/min	$t_{1/2}$ (SD) min
Adult	Struys et al. [27]	1.67 (0.45)	122 (13.2)	NR	NR
	Schnider et al. [28]	1.62 (0.43)	121 (13.8)	NR	NR
	Levitt et al. [29]	1.68 (0.45)	62.4 (7.2)	2.64	NR
	Gepts et al. [30]	1.32 (0.34)	188 (28.2)	1.56 (0.181)	277 (138.5)
	Doufas et al. [31]	1.67 (0.45)	117 (12.6)	2.64 (0.17)†	87.3**
Children	Jones et al. [32]	0.69 (0.25)	172 (29.4)	1.1 (0.18)	209 (26.3)
	Murat et al. [33]	0.36 (0.12)	133.8 (15)	0.57*	124.2**
	Saint-Maurice et al. [34]	0.51 (0.13)	152 (22.2)	0.59	383.6**
Infant	Sepulveda et al. [35]	0.25 (0.11)	136 (20.4)	NR	NR
	Raoof et al. [36]	0.19 (0.11)	179 (20.4)	0.41 (0.01)	100 (31)
Neonate	Allegaert et al. [37]	0.049 (0.02)	161.4 (19.8)	0.034 (0.0093-0.2)	214**
	Allegaert et al. [38]	0.048 (0.02)	251 (49.2)	NA	NA

*SD: standard deviation; CL: clearance; $t_{1/2}$: elimination half-life; †: L/min unit assumed from the study; *: no SD reported; **: half-life was calculated from steady state clearance and volume of distribution reported in the study using the half-life (min) = 0.693 x (Volume of distribution (L) / Clearance (L/min)) equation.*

Table S4. Predicted clearance of propofol across age groups

Age group	CL (SD) L/min
**Neonates**	0.05 (± 0.02)
**Infants**	0.23 (± 0.11)
**Children**	0.82 (± 0.35)
**Adolescents**	1.58 (± 0.50)
**Adults**	1.66 (± 0.45)

data represent mean values following Robert model in adult and Morse model in children SD: standard deviation

Table S5. Mean CL (L/min) with normal, or reduced cardiac output (CO) conditions

	Neonates	Infants	Children	Adolescents	Adults
**Normal CO**	0.05	0.23	0.82	1.58	1.66
**20% ↓CO**	0.05	0.21	0.73	1.40	1.45
**30% ↓CO**	0.04	0.20	0.68	1.29	1.34
**40% ↓CO**	0.04	0.18	0.62	1.17	1.21
**50% ↓CO**	0.04	0.16	0.55	1.04	1.06

CO: cardiac output
